# Circadian Disruption Changes Gut Microbiome Taxa and Functional Gene Composition

**DOI:** 10.3389/fmicb.2018.00737

**Published:** 2018-04-13

**Authors:** Jessica A. Deaver, Sung Y. Eum, Michal Toborek

**Affiliations:** Department of Biochemistry and Molecular Biology, University of Miami Miller School of Medicine, Miami, FL, United States

**Keywords:** circadian disruption, gut microbiota, intestinal barrier integrity, metatrascriptomics, microbiome-host interactions

## Abstract

Disrupted circadian rhythms and alterations of the gut microbiome composition were proposed to affect host health. Therefore, the aim of this research was to identify whether these events are connected and if circadian rhythm disruption by abnormal light–dark (LD) cycles affects microbial community gene expression and host vulnerability to intestinal dysfunction. Mice were subjected to either a 4-week period of constant 24-h light or of normal 12-h LD cycles. Stool samples were collected at the beginning and after the circadian rhythm disruption. A metatranscriptomic analysis revealed an increase in *Ruminococcus torques*, a bacterial species known to decrease gut barrier integrity, and a decrease in *Lactobacillus johnsonii*, a bacterium that helps maintain the intestinal epithelial cell layer, after circadian rhythm disruption. In addition, genes involved in pathways promoting host beneficial immune responses were downregulated, while genes involved in the synthesis and transportation of the endotoxin lipopolysaccharide were upregulated in mice with disrupted circadian cycles. Importantly, these mice were also more prone to dysfunction of the intestinal barrier. These results further elucidate the impact of light-cycle disruption on the gut microbiome and its connection with increased incidence of disease in response to circadian rhythm disturbances.

## Introduction

Circadian rhythms are an endogenous process that regulates the timing of numerous physiological functions (Green et al., 2008; Asher and Schibler, 2011; Eckel-Mahan and Sassone-Corsi, 2013). The suprachiasmatic nucleus (SCN) is the “master clock” primarily responsible for coordinating and synchronizing daily biological rhythms (Asher and Sassone-Corsi, 2015). Approximately 20,000 neurons compose the SCN, which maintains “peripheral clocks” to a 24-h period (Eckel-Mahan and Sassone-Corsi, 2013; Asher and Sassone-Corsi, 2015). Although these clocks function without external input, environmental cues tend to influence circadian rhythms (Asher and Sassone-Corsi, 2015). In particular, light–dark (LD) cycles have impact on the circadian biological clock (Eckel-Mahan and Sassone-Corsi, 2013; Covassin et al., 2016). Circadian rhythms strongly control metabolic processes and inflammation responses, and exposure to light at night can disrupt the timing of these processes (Green et al., 2008; Asher and Schibler, 2011; Eckel-Mahan and Sassone-Corsi, 2013; Fonken et al., 2013; Thaiss et al., 2014; Voigt et al., 2016). With rising disruption of LD cycles in human populations due to night-shift work, excessive use of artificial light, or travel across different time zones, there is increasing concern for the effects of circadian rhythm disruption on susceptibility to disease. In fact, LD cycle alterations have been linked to metabolic imbalances, such as weight gain, and altered inflammation processes (Eckel-Mahan and Sassone-Corsi, 2013; Fonken et al., 2013, 2014).

Further interest has been taken in studying the link between changes and alterations of gut microbiota composition due to variable feeding patterns in response to LD cycle dysregulation (Asher and Sassone-Corsi, 2015; Voigt et al., 2016; Kaczmarek et al., 2017) as compelling evidence indicates that gut microflora signal to the circadian clock (Marcinkevicius and Shirasu-Hiza, 2015; Cui et al., 2016; Paulose et al., 2016; Rosselot et al., 2016). There are approximately 10^10^–10^12^ bacterial species/g in the human intestine (Hakansson and Molin, 2011). Microbial gut colonizers contribute to host adaptation to various environmental cues and diets, nutrient metabolism, and immunity induction (Lozupone et al., 2012; Caricilli et al., 2014). These functions ultimately help to maintain an intestinal homeostasis. Consequently, disturbances of the gut microbiome have been linked to altered immune responses and intestinal inflammatory diseases triggered by disrupted barrier function (Caricilli et al., 2014). It was demonstrated that diseases correlated with alterations of circadian rhythms tend to be impacted by inflammatory processes. At the same time, changes in biological clock functions prompt increases in pro-inflammatory intestinal bacterial abundance, a decrease in anti-inflammatory intestinal bacterial abundances, and impeded intestinal barrier function (Voigt et al., 2016). Interestingly, the intestinal microbiota undergoing diurnal compositional and functional oscillations may also influence host circadian activity (Thaiss et al., 2016); thus, the relationship between the circadian rhythms and gut microbiota appears to be bidirectional (Mu et al., 2016). While the connection between LD cycles, gut microflora, and feeding patterns is relatively well studied, the aim of this study was to further explore whether circadian rhythm disruption solely caused by disturbed LD cycles elicits alterations in intestinal bacterial and functional gene composition.

Recently, metatranscriptomics has gained recognition as a tool for characterizing the microbiome (Bashiardes et al., 2016). This technique recovers total genetic material from an environmental sample, which then allows development of gene expression profiles from culturable or unculturable bacteria (Hakansson and Molin, 2011; Bashiardes et al., 2016). A complete gene profile results in a wider composition analysis to characterize bacterial identity and actively expressed genes in the intestinal microbial community (Bashiardes et al., 2016). Therefore, this study used metatranscriptomic analysis to ascertain whether abnormal LD cycles influence gut microbiome composition and function.

## Materials and Methods

### Treatments

All animal procedures were approved by the University of Miami Institutional Animal Care and Use Committee in accordance with National Institutes of Health (NIH) guidelines and performed in accordance with the relevant guidelines and regulations. Male, 12 week old, C57BL/6J mice (Jackson Laboratories) were allowed to acclimatize to the animal facility for 4 weeks with free access to food and water. Mice were housed individually in separate cages throughout the experiment, including acclimatization period. At the beginning of the experiment, stool samples from each mouse were collected to obtain baseline data sets. One group of mice was subject to normal 12-h LD cycles for 4 weeks. The other group was subject to constant 24-h light for 4 weeks. After this period, stool samples were collected from each mouse again to determine the impact of chronodisruption on the gut microflora. During the experiment, mice were kept in separate cages to avoid a cage effect and to correlate the baseline data with the end-point results individually for each mouse. All mice had free access to food and water; however, no feeding time was recorded. Stool samples were stored in -80°C until analysis.

### mRNA Isolation and Purification

Feces (approximately 2 g per sample) were collected into the Eppendorf tubes at the beginning and end of experiment and stored at -80°C until RNA isolation was performed. The MoBio PowerSoil Total RNA Isolation Kit (Qiagen, Germantown, MD, United States) was used for total RNA isolation from mouse feces according to the instruction provided by the manufacturers and as described recently (Hampton-Marcell et al., 2013). Additional DNA removal treatment was performed by adding 0.1 volume 10× Turbo DNase buffer and 1 μl Turbo DNase (both part of Turbo DNA-Free Kit, Life Technologies) for 30 min at 37°C. Then, 0.1 volume of resuspended DNase Inactivation Reagent was added, the samples were incubate for 5 min at room temperature, mixed, and centrifuged at 10,000 × *g* for 1.5 min. The obtained RNA was then transferred to a new tube.

### Library Preparation, RNA-Seq, and Profiling Methods

In the next step, rRNA was removed using the ScriptSeq Complete Kit for Bacteria (Epicentre). The kit removes sequences found in bacterial–ribosomal RNA with 99% efficiency working within a range of 100 ng to 5 μg of total RNA. Using random hexamers, the kit takes advantage of directional, paired-end sequencing using Illumina’s TruSeq Cluster Kit. Bacterial mRNA was next purified using the AMPure RNAClean XP Beads as described earlier (Hampton-Marcell et al., 2013).

RNA fragmentation improves hybridization kinetics and can lead to enhanced signal. Therefore, bacterial mRNA (2–5 μl) was fragmented by incubation with RNA fragmentation solution in the presence of cDNA synthesis primer at 85°C for 5 min in a thermocycler. The reaction was stopped by placing the tubes on ice. Bacterial mRNA was then reversed transcribed with random hexamers for final library generation (Hampton-Marcell et al., 2013) with StarSiq reverse transcriptase, and tagged at 3′ using Script2Seq Terminal-Tagging Premix. cDNA was then purified with AMPure XP beads. Next, di-tagged cDNA was amplified by PCR using primers provided in the ScriptSeq kit and barcodes were added. Each amplified RNA-Seq library was then purified with the AMPure RNAClean XP Beads. Metatranscriptomic sequencing was performed on an Illumina HiSeq 2500 platform. Libraries were pooled and run with 100 base pair paired-end sequencing protocols.

### Raw Data Processing and Functional Analysis

Kraken, as described by Wood and Salzberg (2014), was used to filter out host sequences from the total shotgun sequences recovered using exact alignments from mouse, mm10, reference genome sequence. Trimmomatic (Bolger et al., 2014) was employed to process continuing reads to remove adapter sequences and low-quality sequence ends. Marker genes were identified using 16,000 reference genomes for more than 7,500 unique species. The filtered DNA sequences were mapped against a reference database. This database contained all proteins within the version 75.0 KEGG databases. Diamond (Buchfink et al., 2015) was employed to search translated DNA sequences, and hits were collected that contained at least 20 amino acids with a minimum of 80% sequence similarity. If there was a read matching several proteins, the one with the highest bit score was collected. The KOs collected were matched to their pathways too.

### Diversity Measures

Alpha-diversity measures diversity within a sample. Shannon diversity uses sample richness and relative abundance of the represented operational taxonomic units (OTU) to calculate an alpha-diversity index. Beta-diversity measures dissimilarities between samples. Every profile was inter-compared pair-wise to establish a dissimilarity score, which was stored in a distance dissimilarity matrix. Comparing similar samples results in distance functions generating lower dissimilarity scores. Bray–Curtis dissimilarity was used to calculate abundance-weighted sample pair-wise differences. The principle coordinate analysis (PCoA) method was used to visualize the complex relationship between samples on a two-dimensional ordination plot. Maximizing the linear correlation between the sample-to-sample dissimilarity values and plot distances then allowed positioning of the points relative to each other.

### *Ex Vivo* Intestinal Permeability Assay

A mouse *ex vivo* intestinal loop model was utilized to assess intestinal permeability. Briefly, 5-cm segments of longitudinal ileum were removed and gently rinsed with ice-cold saline. One end of the segment was ligated and filled with 500 μl of FITC-dextran 4 (MW 4 kDa, 2 mg/ml in PBS) (Sigma-Aldrich). The open end of the segment was then tied off with suture. Segments were transferred into tubes containing 5 ml of 1% FBS in PBS and incubated for 45 min at 37°C. FITC-dextran that transversed the intestine was quantified by fluorescence plate reader (SPECTRAMax Gemini EM, Molecular Devices) using 483 nm as excitation and 517 nm as emission wavelengths and normalized for the length of intestinal segment.

### Statistical Analysis

Permutational analysis of variance (PERMANOV) tests were performed directly on the sample-to-sample distance matrix in order to find significant differences among variables. Samples were randomly reassigned in categories and the *p*-value for the test was reported as the fraction of permutations with larger cross-category differences relative to within category differences. Differential gene expression was determined using a negative binomial noise model for the overdispersion and Poisson process intrinsic to the data for microbiome applications as described by McCurdie and Holmes (2013). The DESeq2 package (Love et al., 2014) was used to run this test under default settings. The analysis pipeline included both custom R code (Second Genome, San Francisco, CA, United States) and published methods (Anderson, 2001; McCurdie and Holmes, 2013; Love et al., 2014). The low sample number resulted in a low statistical power; therefore, the alpha value was adjusted and statistically significant values were considered those with a Benjamini–Hochberg procedure FDR-corrected *p*-value < 0.1.

## Results

### Variation in Microbiome Composition

**FIGURE 1:**
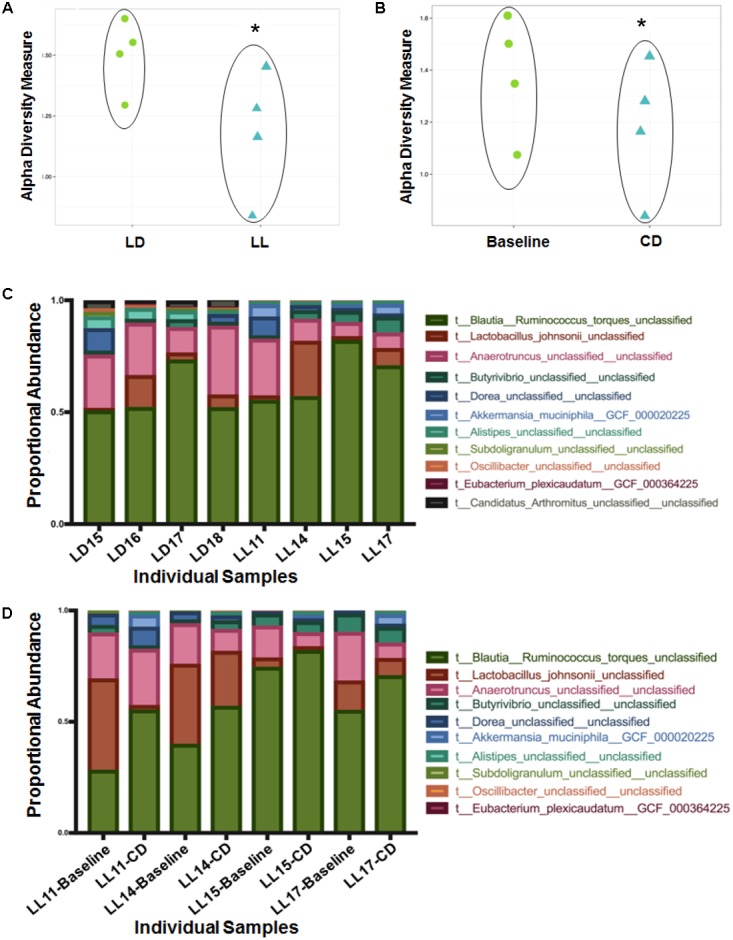
Microbial diversity measured by the Shannon Diversity index for LD versus LL after 4 weeks of circadian rhythms disruption **(A)** and for baseline versus circadian rhythms disruption **(B)** shows distinction in alpha diversity between the comparison groups. ^∗^A significant difference determined by Wilcoxon rank sum test *p*-value 0.057 given alpha 0.1 in the alpha-diversity between the two groups. Taxa abundance represented by strain (composing >0.01% of the sample) for LD versus LL at 4 weeks of circadian rhythms disruption **(C)** and for baseline versus circadian rhythms disruption **(D)**. LD, 12-h light/dark cycles; LL, 12-h light/light cycles.

There was no difference in α- or β-diversity between normal LD cycles and circadian rhythm disrupted (light/light, LL) groups in the baseline measurement (data not shown). After 4 weeks of circadian disruption, Shannon diversity measurements showed a significant microbial α-diversity difference between the LL and LD groups. Indeed, there was a significant difference determined by Wilcoxon rank sum test *p*-value 0.057 given alpha 0.1 in the alpha-diversity between the two groups (**Figure 1A**). Covariate significance analysis revealed that microbial taxa also contributed to β-diversity between these experimental groups (**Table 1**). Comparing the baseline microbial community composition to that after 4 weeks of circadian disruption, no variation in α- or β-diversity was observed (**Figure 1B**).

**Table 1:** Covariate significance.

Variable	Classes	Taxon	Gene	Pathway
Baseline vs. CD	Baseline, CD	0.198	**0.026**	**0.073**
LD vs. LL T1	LD, LL	0.905	0.785	0.664
LD vs. LL T2	LD, LL	**0.087**	**0.034**	0.157

*Significant contribution of each variable to beta-diversity of the samples was determined by performing PERMANOVAs using distance matrices. Significant dissimilarity scores are <0.1 (bolded values). CD, circadian disruption; LD, 12-h light–dark cycles; LL, 12-h light–light cycles; T1, time 1, baseline; T2, time 2, 4 weeks after baseline*.

Changes in microbiome composition taxa were viewed at the species level because there were no changes at the phyla and family level. Direct comparisons at the genus levels were not made. *Ruminococcus torques* were generally more abundant after circadian rhythm disruption when compared to the baseline composition and to the normal LD group microbiome (**Figures 1C,D**). An average percentage abundances of *R. torques* at the baseline was 42% compared to 64% following circadian disruption. In contrast, *Lactobacillus johnsonii* were generally less abundant after circadian rhythm disruption; 22.4% at the baseline compared to 8.79% at the end of experiment (**Figures 1C,D**). *Eubacterium plexicaudatum* and *Subdoligranulum* were not present after 4 weeks of circadian disruption; however, they were present at low abundance in the baseline composition and in the normal LD group microbial community (average 0.11% for *E. plexicaudatum* and 0.24% for *Subdoligranulum*) (**Figures 1C,D**). The described changes in the percentage abundances of the affected species (*R. torques*, *L. johnsonii*, *E. plexicaudatum*, and *Subdoligranulum*) were more in terms of trends as a small sample size precluded these changes to be significant.

### Variation in Functional Genes

**FIGURE 2:**
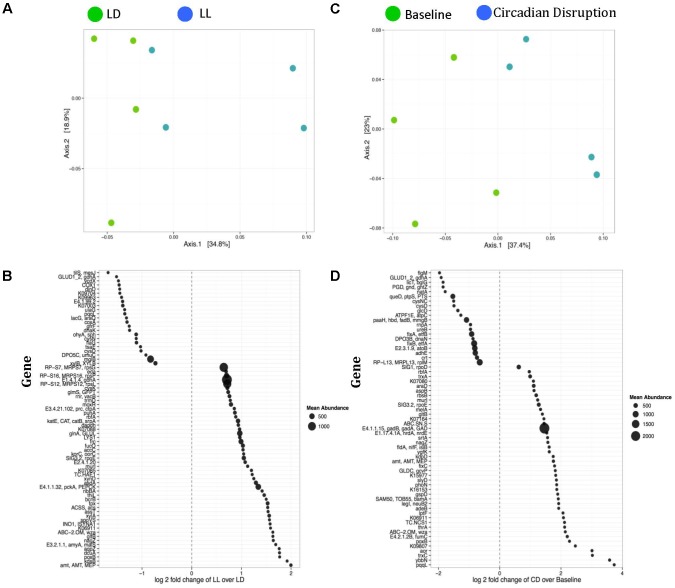
Weighted ordination plots of functional genes show a clear distinction between the LD and LL groups after 4 weeks of circadian rhythms disruption **(A)** and between baseline and circadian rhythms disruption **(C)**. These plots illustrate the dimensional reduction of the Bray–Curtis distance between samples, using the PCoA ordination method. In PCoA, the axes are eigenvectors that separate the samples in high-dimensional space. Two vectors were selected that explain the most variation in the samples. A PERMANOVA using distance matrices was performed for each variable of interest to determine if they significantly contributed to the beta-diversity of the samples. There were 72 differentially expressed genes between the LD and LL groups after 4 weeks of circadian rhythms disruption **(B)** and 63 differentially expressed genes between the baseline and circadian rhythms disruption **(D)**. LD, 12-h light/dark cycles; LL, 12-h light/light cycles.

Weighted ordination plots revealed a clear distinction between gene expression of the normal LD group and the circadian rhythm disrupted group after 4 weeks disruption (**Figure 2A**). A PERMANOVA using distance matrices was performed for each variable of interest to determine if they significantly contributed to the beta-diversity of the samples. There were 72 functional genes differentially expressed between these groups (**Figure 2B**). Weighted ordination plots also reveal a clear distinction of gene expression before and after circadian rhythm disruption (**Figure 2C**), with 63 functional genes differentially expressed (**Figure 2D**). Our further analysis focused on selected genes involved in host metabolism.

### Genes Involved With Microbiome–Host Interactions

**FIGURE 3:**
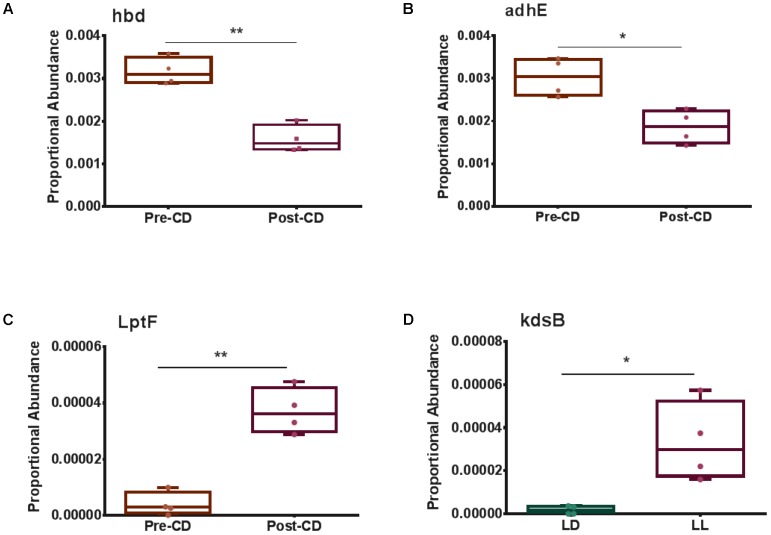
Box and whisker plots illustrating differential gene expression of **(A)** 3-hydroxybutyrl-CoA dehydrogenase, *hbd*; **(B)** Acetaldehyde dehydrogenase, *adhE*; **(C)** Lipopolysaccharide export permease protein, *LptF*; and **(D)** 3-deoxy-manno-octulosonate cytidylyltransferase, *kdsB*. Boxes show minimum to maximum and individual data points. LD, 12-h light/dark cycles; LL, 12-h light/light cycles; CD, circadian disruption. ^∗^*p* < 0.05; ^∗∗^*p* < 0.01.

Selected genes involved with the metabolism and transportation of key metabolites for Microbiome–Host Communication were differentially expressed. For example, 3-hydroxybutyrl-CoA dehydrogenase, *hbd*, was significantly downregulated post circadian rhythm disruption (**Figure 3A**). Acetaldehyde dehydrogenase, *adhE*, showed a strong tendency to be significantly lower (FDR-corrected *p*-value < 0.1) after circadian rhythm disruption (**Figure 3B**). Lipopolysaccharides (LPS) export system permease protein, *LptF*, was expressed significantly higher after circadian rhythm disruption than at baseline (**Figure 3C**). 3-Deoxy-manno-octulosonate cytidylyltransferase, *kdsB*, was also expressed significantly higher in the circadian disrupted group than the normal LD group (**Figure 3D**).

### Circadian Rhythm Disruption Causes Mouse Intestinal Barrier Dysfunction

**FIGURE 4:**
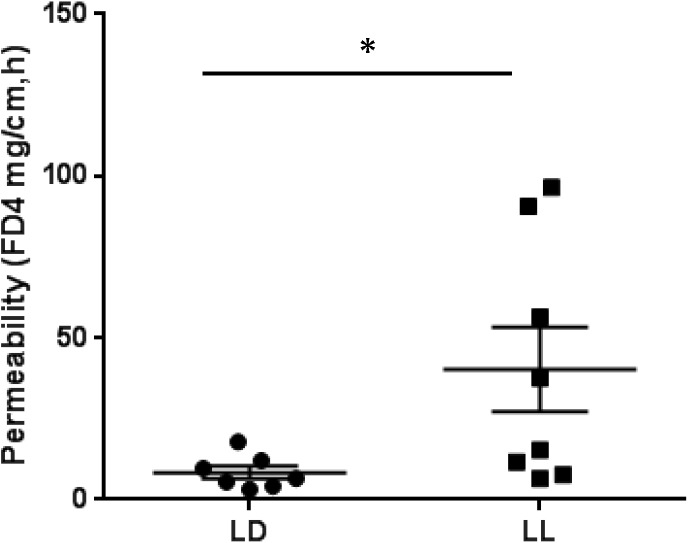
FITC-dextran flux across the intestinal wall of mice exposed to normal LD cycling or constant 24-h light (LL) for 4 weeks; *n* = 7 for LD group and *n* = 8 for LL group. ^∗^*p* < 0.05.

To address whether circadian rhythm disruption can cause the barrier dysfunction of intestinal tissue, we employed an *ex vivo* intestinal permeability test using the ileum extracted either from control mice housed under normal LD cycles or from mice maintained in constant light for 4 weeks. We assessed whether intraluminal 4 kD FITC-dextran translocates across the intestinal wall into the extraluminal space. The group of constant light exposure showed significant increase of intestinal permeability to FITC-dextran compared to the group of normal LD cycling (**Figure 4**). From these experiments, we suggest that circadian rhythm disruption causes significant dysfunction of intestinal barrier.

## Discussion

Diversity changes between the circadian disruption and control groups reveal alterations in composition of the gut microflora. The difference in α-diversity between the group maintained under normal LD cycles and the group exposed to constant light suggests that normal light cycles help maintain a higher variety of gut microflora. Indeed, the disparity in β-diversity suggests that disruption of circadian rhythms impacts which species are present in the microbiome. Although these changes are not seen to a significant degree at the baseline and after a 4-week of circadian rhythm disruption in the same group, the covariate significance is much lower between both baseline measurements, suggesting that taxa still contribute to the β-diversity differences.

The observed increase in *R. torques* and decrease in *L. johnsonii* abundances in the mice subjected to abnormal light cycles may affect intestinal health and contribute to the observed disruption of intestinal barrier functions. Previous studies indicated that *R. torques* may be involved with a decrease in gut barrier integrity (Cani, 2014; Rajilic-Stojanovic and de Vos, 2014). This bacterium is a potent mucus degrader, and its increased abundance has been associated with elevated blood triglycerides levels and irritable bowel syndrome; probiotic consumption lowers its presence (Rajilic-Stojanovic and de Vos, 2014). In contract, *L. johnsonii* is known to be a beneficial bacterium promoting host health. Cell surface proteins of *L. johnsonii* are thought to stimulate immune cells leading to immunomodulation (Pridmore et al., 2004). They have also been shown to induce heat shock protein (HSP) generation (Liu et al., 2015). HSP proteins perform vital housekeeping processes to maintain the intestinal epithelial monolayer, therefore, protecting against pathogens and potential toxic compounds (Liu et al., 2014, 2015). Thus, this noticeable increase in *R. torques* and decrease in *L. johnsonii* reveals a disadvantageous change in the bacterial community post-circadian rhythm disruption.

We also observed lack of *E. plexicaudatum* and *Subdoligranulum* after circadian rhythm disruption compared to the baseline. These changes can also affect the host health as both are butyrate-producing bacteria (Abbasi, 2009; Eckel-Mahan and Sassone-Corsi, 2013). Butyrate is a short-chain fatty acid (SCFA) responsible for stimulating host G-protein-coupled receptors to regulate release of insulin and glucagon, providing energy for host cells, and regulating mucosal gene expression to maintain gut barrier integrity (Abbasi, 2009; Thomas et al., 2014; Boulange et al., 2016; Wong et al., 2016). Butyrate producers contribute to beneficial responses that enhance the intestinal health, and low *Subdoligranulum* abundance has been correlated with increased incidences of inflammatory bowel disease (Thomas et al., 2014). These bacterial species were only found in the groups not subjected to circadian disruption, further supporting the notion that abnormal LD cycles promote degradation of beneficial intestinal microbial communities. The shifts in microbiome taxa composition after circadian disruption suggest declining intestinal health and a higher susceptibility to inflammation and decreased gut barrier integrity in the host. While the exact mechanisms of these changes are not fully understood; there may be a consequence of disruption of central or peripheral (e.g., intestine and liver) circadian machinery and/or due to central/peripheral circadian misalignment.

Although alterations in taxa were apparent, the variations in gene expression reveal functional changes that may contribute to increased susceptibility to disease in hosts with disrupted circadian rhythms. Comparison between the baseline and after circadian rhythm disruption revealed 63 differentially expressed genes. Similarly, 72 genes were differentially expressed between the circadian disrupted and normal light groups. Various metabolism genes, ABC transporter genes, flagellar assembly genes, and two-component system genes were differentially expressed, suggesting that circadian rhythm disruption contributes to functional shifts that change the way symbiotic bacteria interact with the host. Notably, the expression of genes related to butyrate production, indole and indole derivative generation, and LPS production and transport was altered.

One of the key enzymes in bacterial butyrate synthesis is 3-hydroxybutyrl-CoA dehydrogenase (*hbd*) (Vital et al., 2014). Microbiome bacteria produce butyrate from undigested carbohydrates through the acetyl-CoA production pathway (Hammer et al., 2007). Levels of *hbd* were significantly downregulated after circadian rhythm disruption, which may impede host health because butyrate functions to provide energy for intestinal epithelial cells and to bolster inhibition of inflammation and carcinogenesis (Abbasi, 2009; Thomas et al., 2014; Boulange et al., 2016; Wong et al., 2016). Acetaldehyde dehydrogenase (*adhE*), which participates in indole and indole-derivative formation via aromatic compound degradation pathways, was also significantly downregulated after circadian rhythm disruption. Indole and its derivatives play roles in activating anti-inflammatory processes and protecting the intestinal barrier by up regulating junction proteins and down regulating TNF-α, a pro-inflammation signaling molecule (Devaraj et al., 2013). Down regulation of these genes suggest a decrease in production of butyrate and indole or its derivatives, and therefore a decline in beneficial functions of these metabolites.

Upregulation of genes responsible for LPS synthesis and transportation suggests an increase in host exposure to this deleterious compound. LPS is a component of Gram-negative bacteria cell walls and can translocate into the host systemic system via a permeable gut barrier (Hakansson and Molin, 2011; Boulange et al., 2016). The immune responses triggered by LPS occur when it binds the CD14 receptor on macrophages, followed by activation of inflammatory effectors (Hakansson and Molin, 2011; Boulange et al., 2016). High systemic concentrations of LPS cause endotoxemia (Hakansson and Molin, 2011; Boulange et al., 2016). After circadian rhythm disruption, LPS export permease protein (*LptF*), an ABC transporter, was significantly upregulated compared to baseline. This transporter translocates LPS from the inner to the outer bacterial cell membrane; thus, its increased expression suggests higher LPS production and utilization. In addition, 3-deoxy-manno-octulosonate cytidylyltransferase (*kdsB*), a gene involved in LPS synthesis, was also significantly upregulated in the microbiome community of the disrupted LD cycle group as opposed to controls. This upregulation suggests an upsurge in LPS production, which may result in LPS entering host circulation and increased systemic inflammation. We consider the observations that chronodisruption upregulated the microbial genes controlling LPS synthesis as the most important finding of the present study, which merit further mechanistic studies. Indeed, chronic inflammation induced by elevated LPS levels can be an underlying mechanism of several systemic diseases, ranging from obesity to neuro-inflammatory responses in the brain (Cani et al., 2007; Qin et al., 2007). LPS can be one of the most critical elements of the gut–brain axis negatively affecting the brain in response to the alterations of gut microbial communities and intestinal integrity. These observations can provide at least partial explanation of why people subjected to circadian rhythm disruption are potentially more prone to the development of metabolic diseases and cancer (Wang et al., 2011).

The increase in intestinal permeability observed after circadian disruption also suggests a decline in host health. Maintaining an intact, appropriately permeable intestinal barrier plays a significant role in selecting between factors that should cross the barrier into the body and those that are harmful (Caricilli et al., 2014). As this barrier, composed of mucous and an epithelial cell monolayer sealed together by tight junction proteins, loses integrity, damaging factors, such as LPS or pathogenic bacteria, are more likely to translocate across the intestinal wall (Caricilli et al., 2014).

## Conclusion

The present study reveals changes in bacterial composition and functional gene expression as well as impaired intestinal integrity correlated with circadian rhythm disruption due to abnormal LD cycles. The decrease in diversity measures, changes in bacterial community composition, and alterations in key gene expressions suggest that circadian rhythm disruptions result in induction of bacterial factors that can negatively impact intestinal homeostasis and host health. Specific factors responsible for these effects include decreased levels of bacteria considered to be beneficial, combined with a rise in bacterial species known to impede gut barrier integrity as well as with an increase in available LPS and decrease in butyrate levels. Enhanced intestinal permeability further supports the perceived connection between abnormal LD cycles and declining host intestinal homeostasis.

## Author Contributions

JD analyzed the results, prepared figures, and wrote the manuscript. SE designed the study and performed the experiments. MT designed the study and provided the logistic and grant support to perform the experiments and analyzed the results. All authors reviewed the manuscript.

## Conflict of Interest Statement

The authors declare that the research was conducted in the absence of any commercial or financial relationships that could be construed as a potential conflict of interest.
